# Level and Noise Sources in the Neonatal Intensive Care Unit of a Reference Hospital

**DOI:** 10.17533/udea.iee.v38n3e13

**Published:** 2020-11-11

**Authors:** Alma Damaris Hernández-Salazar, Josefina Gallegos-Martínez, Jaime Reyes-Hernández

**Affiliations:** 1 Nursing Degree, Specialist. Email: almadhs29@hotmail.com almadhs29@hotmail.com; 2 Nursing Degree, PhD. Professor and Full-time Researcher. Email: jgallego@uaslp.mx. Corresponding author jgallego@uaslp.mx; 3 Engineer, PhD. Professor and Full-time Researcher. Email: reyes.jaimeh@uaslp.mx reyes.jaimeh@uaslp.mx; 4 Universidad Autónoma de San Luis Potosí, S.L.P., México. Universidad Autónoma de San Luís Potosí Universidad Autónoma de San Luis Potosí S.L.P Mexico

**Keywords:** intensive care units, neonatal, infant, premature, noise measurement, interior design and furnishings, unidades de cuidado intensivo neonatal, recién nacido prematuro, medición del ruido, diseño interior y mobiliario., unidades de terapia intensiva neonatal, recém-nascido prematuro, medição de ruído, decoração de interiores e mobiliário.

## Abstract

**Objective.:**

Determine the level of environmental and periauricular noise in preterm babies and identify the sources generating noise in the Neonatal Intensive Care Unit -NICU- of a reference hospital in San Luis Potosí, Mexico.

**Methods.:**

Cross-sectional and analytic study of the measurement of the level of environmental noise in five critical areas of the NICU, according with the method of measurement of noise from fixed sources by the Mexican Official Norm and periauricular at 20 cm from the preterm patient’s pinna. The measurements were carried out during three representative days of a week, morning, evening and nocturnal shifts. A STEREN 400 sound level meter was used with 30 to 130 dB range of measurement and a rate of 0.5 s.

**Results.:**

The average level of periauricular noise (64.5±1.91dB) was higher than the environmental noise (63.3±1.74 dB) during the days and shifts evaluated. The principal noise sources were activities carried out by the staff, like the nursing change of shift and conversations by the staff, which raised the level continuously or intermittently, operation of vital support equipment (alarms) and incidences (clashing of baby bottles and moving furnishings) produced sudden rises of noise.

**Conclusions.:**

Environmental and periauricular noise in NICU exceeds by two and almost three times the 45 dB during the day and 35 dB at night from the norm in hospitals. It is necessary to implement permanent noise reduction programs to prevent sequelae in the preterm infant and professional burnout in the nursing staff.

## Resumen

**Objetivo.:**

Determinar el nivel de ruido ambiental y periauricular en prematuros e identificar las fuentes generadoras de ruido en la Unidad de Cuidados Intensivos Neonatales -UCIN- de un hospital de referencia de San Luis Potosí, México.

**Métodos.:**

Estudio transversal y analítico de la medición del nivel de ruido ambiental en cinco áreas críticas de la UCIN de acuerdo al método de medición de ruido de fuentes fijas de la Norma Oficial Mexicana y periauricular a 20 cm del pabellón auricular del prematuro. Las mensuraciones se realizaron en tres días representativos de una semana, turnos matutino, vespertino y nocturno. Se utilizó un sonómetro STEREN 400 con rango de medición de 30 a 130 dB y una frecuencia de 0.5 s.

**Resultados.:**

El promedio del nivel de ruido periauricular (64.5±1.91dB) fue mayor que el ambiental (63.3±1.74 dB) en los días y turnos evaluados. Las principales fuentes de ruido fueron las actividades realizadas por el personal como entrega de turno de enfermería y conversación del personal que elevó el nivel de manera continua o intermitente, el funcionamiento del equipo de soporte vital (alarmas) e incidencias (choque de biberones y mover mobiliario) produjeron elevaciones súbitas de ruido.

**Conclusiones.:**

El ruido ambiental y periauricular en UCIN sobrepasa al doble y casi al triple los 45 dB en el día y 35 dB por la noche de la normativa en hospitales. Es necesario implementar programas permanentes de reducción de ruido para prevenir secuelas en el prematuro y desgaste profesional en el personal de enfermería.

## Resumo

**Objetivo.:**

Determinar o nível de ruído ambiental e periatrial em bebês prematuros e identificar as fontes geradoras de ruído na Unidade de Terapia Intensiva Neonatal -UTIN- de um hospital de referência em San Luis Potosí, México.

**Métodos.:**

Estudo transversal e analítico da medição do nível de ruído ambiental em cinco áreas críticas da UTIN de acordo com o método de medição de ruído de fontes fixas do Padrão Oficial Mexicano e periauricular a 20 cm da orelha do prematuro. As medições foram realizadas em três dias representativos da semana, turnos matutinos, vespertino e noturno. Foi utilizado um medidor de nível de som STEREN 400 com faixa de medição de 30 a 130 dB e frequência de 0.5 s.

**Resultados.:**

O nível médio de ruído periatrial (64.5±1.91 dB) foi superior ao ruído ambiente (63.3±1.74 dB) nos dias e turnos avaliados. As principais fontes de ruído foram as atividades realizadas pela equipe, como entrega do plantão de enfermagem e conversas com a equipe que aumentavam o nível de forma contínua ou intermitente, o funcionamento do equipamento de suporte de vida (alarmes) e incidentes (choque de mamadeira e movimentação de móveis) produziu picos repentinos de ruído.

**Conclusões.:**

O ruído ambiental e periatrial na UTIN é mais do que o dobro e quase o triplo dos 45 dB diurnos e 35 dB noturnos dos regulamentos dos hospitais. Faz-se necessária a implantação de programas permanentes de redução de ruído para prevenir sequelas em prematuros e esgotamento profissional na equipe de enfermagem.

## Introduction 

Preterm birth is a global public health problem; it is estimated that approximately 15-million preterm neonates are born annually, which translates into a little more than one for every ten children, a number on the increase.(1) In 2018 in Mexico, 2,162,535 children were born and 48,145 in the state of San Luis Potosí,(2) approximately between 5% and 18% corresponded to preterm births.(1) Specifically, in the reference hospital of the present study, a prematurity prevalence of 11.9% was reported from the retrospective analysis of 5,462 births from October 2014 to September 2015.(3) Prematurity in many cases makes hospitalization necessary for prolonged periods in the neonatal unit (NU), given that the preterm child has problems with feeding, temperature regulation, as well as respiratory and infectious problems (3) and are administered specialized treatments due to the clinical situation and support for pulmonary maturation, as well as treatments with aminoglycoside antibiotics, conditions that prolong the hospital stay and which consequently overexpose them to different harmful stimuli for their hearing development, especially due to noise levels > 45 dB, which is the limit recommended by the American Academy of Pediatrics (AAP).(4) Association has been observed between exposure to noise ≥ 60 dB with the effect of ototoxic agents, like aminoglycosides that can damage the ciliated cells of the ear and cause repetitive toxic reactions in the structures of the internal ear due to mechanisms of mutations in the mitochondrial deoxyribonucleic acid.(5)

Hearing deficit in neonates is between 0.1% and 0.6%, in those discharged from the Neonatal Intensive Care Unit (NICU) between 2% and 4%, and for preterm births it can have a prevalence up to 10%.(6) Exposure to noise at high levels produces physiological disorders, like high blood pressure, apnea, or bradycardia, and implies increased oxygen consumption with alterations in saturation, which increases the probability of new episodes of apnea, bradycardia, and diminished amount of calories available for the child’s growth. Sleep disorders can occur due to its discontinuity, especially in preterm patients, which is contrary to the intrauterine environment in which they remained asleep 80% of the time. The fetus perceives and reacts to low-frequency sound, processes the tone and intensity of the human voice in protected manner, which ensures optimal development of the peripheral auditory system and of the neocortical and cochlear relationship, lower gestational age indicates greater compromise of cerebral and sensory development of the preterm neonate, given the neonate’s difficulty to select information from sound received and their inhibitory controls are more susceptible to the effects of the environment, not being able to distinguish the maternal voice from other female voices, which can affect their emotional development.(4,7,8)

The premature patients in the NICU are subjected to stress due to high-intensity sounds derived from equipment and staff (alarms, ventilators, telephones, and conversation) and other intense noises of short duration and at irregular intervals, which is why it is crucial to maintain a stable physiological state especially during this critical period for neurodevelopment.(7,8) Studies on noise levels in the NU have applied environmental measurements, as is the case of the hospital in the present study that, according with the measurements carried out in 1996 in six areas (the NICU was not included) and in four different schedules for three minutes during seven days; noise levels > 59 dB were observed, the critical moments of noise were at 07:00 h during change of shift and at 11:00 h, time of maximum activity in the pediatric ward, adult ICU and hallways, where the noise exceeded 70 and 80 dB.(9)

A study evaluating the modifications of noise level in the NICU in two wards (A and B) before and after the “quiet hour” showed that prior to the intervention in both wards, noise exceeded 70 dB and after the quiet hour the noise level dropped close to 20 dB in both wards; although the authors express that only during the quiet period was said reduction observed. It should be highlighted that for measurements inside the incubators, the microphone of the sound level meter was placed 20 cm from the neonate’s pinna, given that it is the distance at which the neonate perceives better,(10) a criterion considered for application in the present study with the difference that it was carried out in preterm infants in servo cradles.

High noise levels in NICU not only affect the neonates hospitalized, harmful effects have also been reported in the nursing staff who remain during complete shifts in the NICU. The effects include physiological alterations, like increased blood pressure and heart rate, as well as headaches. The noisy environment also contributes to professional burnout and irritability of the staff; these physiological and mood alterations produce problems in the performance of the nursing staff and gives way to a greater number of errors and accidents, the prevalent situation is that nursing is not trained to apply measures to prevent excessive noise and may even “become used” to the environment and not perceive the noise stimuli.(11,12)

This situation makes it essential to identify the intensity and factors that generate noise in the NICU, which permits modifying towards a favorable environment for the good development of neonates at risk, especially those born preterm, as well as the performance of the nursing staff. Due to the aforementioned, the study sought to determine the level of environmental and periauricular noise in preterm babies and identify the sources generating noise in the Neonatal Intensive Care Unit of a reference hospital.

## Methods

Design. A cross-sectional and analytic study was conducted in the NICU of a reference hospital located in the city of San Luis Potosí, Mexico. The study had as unit of observation the intensity of periauricular noise in premature patients and intensity of environmental noise in the NICU.

**Figure 1:**
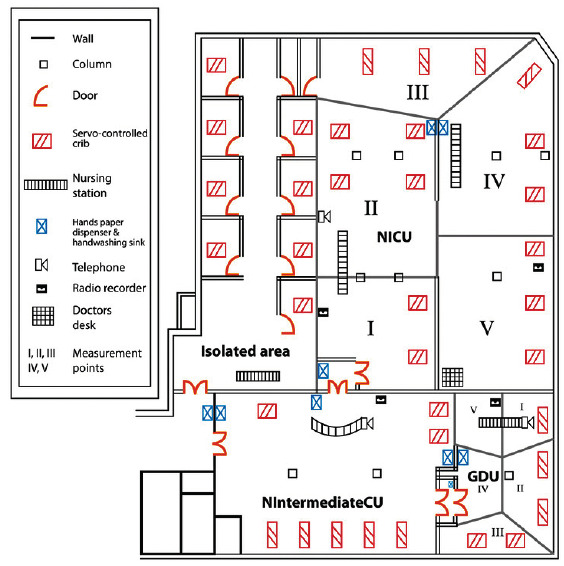
Intensive Care Unit of a reference hospital, San Luis Potosí, S.L.P., México

Place of study. The NICU is located in the NU of the hospital and has a floor area of 15.5 x 12.5 x 3 m with capacity for 28 patients; besides the NICU, the NU has the Newborn Intermediate Care Unit (UCIREN, for the term in Spanish), Growth and Development Unit (GDU) and Isolated Unit (not available at the moment of study). The GDU was used for the pilot test and the NICU for the definitive collection, as observed in Figure 1.

Human resources in the NICU. The morning shift has six to seven nurses, two adjunct physicians, two medical residents, an intern medical student, and one to five medical students and others from the health area, a manager, a radiologist, and a social worker. The evening and night shifts also have six to seven nurses, an adjunct physician, an intern medical student, and one to five external medical students. There is a greater number of staff from Monday to Friday (morning and evening shifts), during nursing change of shift, the morning medical visit, and visits from relatives, Figure 1 shows the distribution of the neonate ward.

Designed by: Mr. Miguel A. Martínez Martínez and Lic. DG Rafael Jeshua Rivera Gallegos. The floor plan of the neonatal unit is not in scale, it is only representative of the areas that compose it.

Sample and Sampling. Premature patients. A non-probability sample and intentional sampling was selected for premature patients due to their vulnerability and risk for the phenomenon studied. The simple comprised nine preterm neonates admitted to NICU and who remained there during the three days evaluated and who fulfilled the inclusion criteria: preterm or small for the gestational age according with the file’s clinical record, whose mothers and/or fathers accepted to participate in the study and signed the informed consent. The patients in the sample were located in servo-controlled thermal cradles and were distributed in the critical measurement areas of the NICU: one patient in areas I, II, III and V, respectively and five patients in area IV. The gestational age of the preterm patients (evaluated through Capurro or Ballard) had a gestation median of 32 ±2.9 weeks (26 to 35.5) and the weight had a median of 1527.78 ±528g at birth (610 to 2,180 g).

Measurement points. Five critical measurement areas were determined. Through equidistant points, (13) representativeness was sought, given that the dimensions of the NICU area - in general - are asymmetrical; it is divided by walls and the space between each patient’s servo cradle is different and was defined in the following manner (Figure 1): I. Entrance to NICU (two servo cradles and heavy transit by staff); II. Intermediate zone (four servo cradles, automatic paper dispenser, wash sink with automatic water jet, and ward telephone); III. Zone away from the entrance (three servo cradles, paper dispenser, wash sink with automatic water jet, and small storage space and light transit by the staff); IV. Intermediate zone (three servo cradles, paper dispenser, nursing control, and radio recorder); and V. Side area at the entrance (three servo cradles, desk for physicians, computer and printer, away from the wash sink and without transit by the staff).

Collection period. The study took three representative days of a work week, thus, Monday and Friday were representative of working days and Sunday was representative of the weekend, given that the behavior of noise levels varies according with the activities, schedules, and days of the week in the NICU. Based on this, schedules were chosen to collect data, thus, during the morning shift from 07:00 to 11:00 h (nursing change of shift, medical visit, inter-consultations, and higher number of staff); during the evening shift from 14:00 to 18:00 h (nursing change of shift and rotation of medical residents, visits from relatives, morning medical visit); and during the night shift from 21:00 to 01:00 h (nursing change of shift, night medical visit).

Data collection. This was carried out by two nursing professionals, one of them (first author) in process of specializing in advanced clinical nursing with emphasis in pediatrics and the other licensed in nursing; both work in the NU of the study hospital. Prior to collecting data through pilot test, training was carried out to handle and calibrate the noise measurement equipment and complement it with computer equipment, establish connections, perform the measurements and data registries, supported by a computer systems engineer. The definitive collection gathered 16,200 registries (one registry every 5 s) of the level of environmental noise from the five critical areas in the NICU, periauricular noise from the nine premature patients, and 90 registry sheets of the noise sources. All the data were used for tabulation in function of the duration of the measurement of only three representative days of a week, given that in other studies the duration was up to several weeks.

Instruments for information collection. (i) General data registry spreadsheet: number of cradle, initials of patient’s name, gestational age and weight at birth, method of evaluation of gestational age, person responsible for the measurement, critical measurement area, date of data collection, time of start and end of measurement; (ii) Checklist from noise-generating sources: designed based on knowledge on the area of study and from the literature review; and (iii) Decibel meteror sound meter (STEREN 400): range of measurement: 30-130 dB, preciseness +2 dB, resolution 0.1 dB, sampling frequency 0.5 s, microphone, amplifier, weighting networks, and a level indicator, all fulfilling the norms by the American National Standards Institute (ANSI) (14) complemented with a Toshiba portable computer, Windows XP Professional emulator system, Sound Level Meter software, RS 232 connection cable with port adaptor to fiber optic USB.

Procedures. *(i) Pilot test.* With prior training of the staff to collect data from the five critical areas pre-established in GDU to fine tune the measurement procedures, a pilot test was conducted one week before (a working day in three shifts) the definite collection. This resulted in modifications only for the registry of noise sources; *(ii) Periauricular measurement.* Through universal norms of hand and equipment asepsis and antisepsis, the sound meter, computer, and microphones were connected, programming the range of measurement from 40 to 90 dB, fast-measurement mode, time range 0.5 s. The Windows XP emulator system was accessed to the Removable Devices option*, Y.C. USA* USB to serial cable connect to host and once activated all these options necessary for recognition of the USB cable by the Windows system, the sound level meter software was accessed and measurement began of the nine premature patients at 20 cm from the pinna (10) for 15 min in each patient during each shift for the three days evaluated. At the end of the 15 min, the *stop* icon was pressed on the software and *off* on the decibel meter. The file was backed up on the icon save and it was labeled according to the start time and day of data collection and, thus, continued until completing the patients from the five predetermined areas. Measurement of environmental noise. In Critical areas I, II, III, IV, V, the sound meter was placed on a tripod at a height of 1.3 m from the floor, according to the norm,(13) the measurement was performed through a semi-continuous measurement for a minimum 15-min period in each area, in each point, and in each shift of the three representative days; (iii) *Noise-generating sources:* each noise generating source was identified and registered during the 15-min period of measurement per shift, coinciding with the loudness peaks, according with the graphic from the Sound Level Meter software on the computer screen.

Data analysis. Categorical data were tabulated and represented through frequencies and percentages; the continuous data through measures of central tendency and dispersion. Comparison of medians was conducted through analysis of variance (ANOVA) statistical test and Student’s t test for related samples, significance was established at *p* ( 0.05.

Ethical aspects. The protocol was submitted to the Ethics and Research Committee in the study hospital and approved (registry 07-14). In addition, the informed consent was obtained signed by the mother and/or father of the preterm patient.

## Results

In all, 16,200 registries were obtained of the environmental level of noise from the five critical areas of the NICU and periauricular noise in the nine premature patients of the sample, which are described ahead for each category.

### Level of periauricular noise per day and shift

The average intensity of periauricular noise (64.5 ±1.91 dB) was higher during the three days and in the three shifts evaluated with respect to the average intensity of environmental noise (63.3 ±1.74 dB), except for Sunday during the evening shift. The level of periauricular noise on the days evaluated behaved differently; in decreasing order, Friday had an average value of 64.8 ±2.3 dB, Sunday of 64.1 ±2.5 dB, and Monday of 63.6 ±1.7 dB. The noise-level behavior with respect to the shifts evaluated was also different, thus, the morning and evening shifts together registered a range from 59.06 to 77.73 dB, exceeding from 14.06 to 32.73 dB (31.2% to 72.7%) with respect to the daytime standard of 45 dB for hospitals and during the night shift it varied from 60.8 to 73.5 dB and exceeded between 25.8 and 38.5 dB (73.7% to 110%), also above the recommendation with respect to nightly 35 dB for hospitals, according with the AAP. (4)

### Level of environmental noise per day and shift

The average intensity of environmental noise was lower (63.3 ±1.74 dB) in the three days and in the three shifts evaluated with respect to the average intensity of periauricular noise (64.5 ±1.91 dB), except for Sunday during the evening shift in which the environmental average surpassed the periauricular average. The level of environmental noise during the days evaluated was different; in decreasing order, it registered on Friday a median of 63.7 ±1.9 dB, Sunday at 63.6 ±2.4 dB, and Monday at 62.6 ±2.0 dB (p ( 0.05). In the three shifts, the level of environmental noise exceeded the recommendations, thus, during the morning and evening shifts together it ranged from 59.2 to 75.01 dB, exceeded between 14.2 and 30.01 dB (31.5% to 66.6%) with respect to the daytime standard of 45 dB for hospitals and during the night shift it varied from 59 to 74.6 dB and exceeded between 24.0 and 39.6 dB (68.5% to 113.1%) above the recommendation with respect to 35 dB at night for hospitals according with the AAP.(4) The comparison between both measurement points, periauricular and environmental, per day and shift evaluated can be observed in Table 1.

**Table 1:** Intensity of periauricular and environmental noise in decibels, according with the day and shift in the Neonatal Intensive Care Unit of a reference hospital

Day and Shift	Periauricular Median (SD)	Environmental Median (SD)	T	DF	***p* - value**
Friday morning	66.4 ±2.8	64.6 ±1.3	16.049	844	**
Sunday morning	64.1 ±2.7	63.4 ±2.6	7.193	867	**
Monday morning	64.7 ±2.2	63.8 ±2.2	11.004	907	**
Friday evening	64.4 ±2.2	63.5 ±2.4	7.322	906	**
Sunday evening	63.1 ±2.8	63.4 ±2.6	-2.466	897	*
Monday evening	63.2 ±3.1	61.7 ±1.8	13.980	922	**
Friday nocturnal	63.7 ±1.7	63.1 ±2.0	6.467	890	**
Sunday nocturnal	65.2 ±1.5	64.0 ±2.1	13.109	897	**
Monday nocturnal	62.9 ±2.3	62.5 ±2.0	3.986	894	**

SD = Standard deviation. t = Student’s t test for related samples. DF = degrees of freedom. Significance *p ( 0.05; ** *p* ( 0.001

### Level of periauricular and environmental noise, according to critical measurement area

With respect to the behavior of the level of periauricular noise in the five critical measurement areas, it was higher in areas I, II, and IV comparatively with the environmental level (p ( 0.05), not so in areas III and V that had similar behavior in noise levels (p ( 0.05). The noise level in the five critical measurement areas of NICU was > 60 dB and, hence, registered higher levels than those of safety required by the AAP norm.(4) Data are presented comparatively by critical measurement area and by points of periauricular and environmental measurement in Table 2. 

**Table 2:** Intensity of periauricular and environmental noise in decibels, according with the critical measurement area in the Neonatal Intensive Care Unit of a reference hospital

Measurement area	Periauricular Median (SD)	Environmental Median (SD)	T	DF	*p-value*
I	64.1 ±2.2	62.7 ±1.7	3.339	16	**
II	64.9 ±1.4	64.1 ±1.7	2.597	15	*
III	64.5 ±1.7	64.7 ±1.3	-0.568	17	-
IV	64.7 ±1.5	63.6 ±1.6	3.741	17	**
V	62.1 ±1.5	61.9 ±1.1	0.490	16	-

SD = Standard deviation. t = Student’s t test for related samples. DF = degrees of freedom. Significance *p < 0.05; ** *p* ( 0.001

### Noise sources

Table 3 shows the noise-generating sources observed during the three days and their respective shifts evaluated; in general, the ranges of noise level varied from somewhat over 60 dB to nearly 100 dB in both periauricular and environmental measurement points. Most of the sources produced sudden noises that exceeded the corresponding norm for transitory noises.(15) On the one hand, sources of transitory sudden noise existed that produced similar noise levels in the points of periauricular and environmental measurement (p ( 0.05), such as movement, opening and closing of opening or closing of furniture and fixtures with an average of 70.1 ±3.9 dB and 68.8 ±2.6 dB periauricular and environmental, respectively, (clashing of baby bottles, drawing of curtains, movement of the cradle’s side door, opening or closing of drawers on the red cart, placement of objects on the nursing control table, on cradles and on Pasteur tables, as well as running water). On the other hand, most of the sources generating sudden and transitory noise contributed to producing a level of periauricular noise higher than the environmental (p ( 0.05), said sources came from care devices and equipment, such is the case of alarms with an average of 70.9 ±5.2 dB and 67.7 ±2.6 dB periauricular and environmental, respectively, (mechanical ventilator, cradle and infusion pumps), not so for monitor alarms (p ( 0.05); varied incidences also contributed, one of them related with the organization and/or activities in the NICU, like sounds from the ward’s telephone and the suction intake, along with crying from patients.

Sources of sudden and transitory noise were observed, which produced higher level of environmental noise compared with periauricular noise (p ( 0.05), some came from accidents, like objects falling to the floor and others from the use of furnishings, like the paper dispenser and dragging tables with 70.8 ±6.7 dB and 68.1 ±2.4 dB, environmental and periauricular, respectively. Sources of continuous noise were also noted produced by constant use of nebulizers, 69.8 ±1.0 dB and 68.8 ±1.9 dB, periauricular and environmental, respectively, and it worth highlighting that there were noise sources from formal and informal human interaction. In the first case, within the care programming in NICU, the change of shift of the nursing staff produced less noise in the periauricular point (68.4 ±2.6 dB) than in the environmental (71.3 ±1.7 dB) (p ( 0.05) and the medical visit, 63.5 ±2.1 dB and 66.3 ±2.5 dB, periauricular and environmental, respectively, without significant difference in the levels of both measurement points (p ( 0.05). The source of informal interaction producing the most continuous noise and a higher level of periauricular noise (68.8 ±3.5 dB) compared with environmental noise (68.4 ±2.6 dB) (p ( 0.05) was conversations by the nursing staff, which was observed during the three days and in almost all the shifts evaluated. 

**Table 3:** Noise intensity in decibels in decreasing order, according to generating source in the NICU of a reference hospital

Noise source	Periauricular (dB)	Environmental (dB)	DF	F	***p* value**
	Median (SD)	Median (SD)			(day/shift/point of measurement)
	(Range)	(Range)			
1. Clash of the bottles	75.9 ±11.4	68.8 ±0.3			
	(69.0 - 96.0)	(68.5 - 69.1)			
2. Alarm from mechanical ventilator	75.2 ±9.6	68.5 ±2.8			
	(65.9 - 96.3)	(61.5 - 75.5)			
			11	241.140	*FEvePer
			26	7.354	*FEveEnv
			26	150.203	**FNocEnv
			11	284.587	*MMorEnv
			26	15.636	**MEvePer
			26	7.153	*MNocEnv
			26	13.976	**SNocEnv
3. Alarm from cradle	75.0 ±5.5	69.3 ±3.5			
	(64.4 - 87.0)	(64.4 - 76.0)			
			20	374.900	*SMorEnv
4. Drawing of curtain	72.7 ±2.4	69.5 ±8.7			
	(69.1 - 74.5)	(61.0 - 78.0)			
5. Cradle side door (movement)	72.6 ±4.7	69.1 ±3.5			
	(66.9 - 78.8)	(64.3 - 78.7)			
6. Alarm from infusion pump	71.4 ±3.9	67.1 ±3.0			
	(66.4 - 82.0)	(64.0 - 77.0)			
			13	13.802	*MEvePer
			14	452.071	*SMorEnv
7. Ward telephone	70.2 ±4.3	68.8 ±2.0			
	(64.9 - 78.8)	(66.8 - 71.8)			
			10	15.131	**FMorPer
			10	42.816	**FNocEnv
			10	14.566	**MEvePer
			10	7.221	*SMorPer
8. Drawers from red cart (opening - closing)	70.1 ±3.3	68.6 ±0.1			
	(67.7 - 72.4)	(68.5 - 68.7)			
9. Nebulizer	69.8 ±1.0	68.8 ±1.9			
	(68.5 - 71.2)	(67.1 - 70.9)			
10. Crying by patient	69.6 ±4.2	67.5 ±2.8			
	(64.9 - 78.0)	(65.2 - 72.0)			
			4	273.750	*FMorPer
			4	905.167	*MNocPer
11. Objects falling to the floor	69.0 ±3.0	71.3 ±7.6			
	(63.4 - 72.6)	(64.4 - 98.0)			
			14	42.997	*FEvePer
			16	273.691	*SEvePer
12. Monitor alarm	68.9 ±6.6	66.7 ±1.9			
	(61.0 - 85.1)	(63.8 - 70.3)			
13. Conversation by staff	68.8 ±3.5	68.4 ±2.6			
	(61.0 - 78.2)	(63.2 - 74.1)			
			30	4.299	*FMorPer
			25	709.171	**FEvePer
			25	89.879	*FNocEnv
			30	3.279	*MMorEnv
			30	3.646	*MNocPer
			30	3.007	*SMorEnv
14. Nursing change of shift	68.4 ±2.6	71.3 ±1.7			
	(64.8 - 73.0)	(69.5 - 73.1)			
			10	246.483	*FEvePer
			10	313.046	*MMorEnv
15. Placement of objects in control by nursing	68.2 ±3.6	66.9 ±1.3			
	(63.7 - 71.9)	(65.6 - 68.2)			
16. Placement of objects on cradle	68.0 ±0.8	72.5 ±1.0			
	(67.2 - 68.7)	(71.5 - 73.5)			
17. Paper dispenser	68.0 ±2.4	69.8 ±5.0			
	(63.3 - 71.0)	(65.7 - 86.5)			
			13	256.036	*MMorEnv
18. Placement of objects on Pasteur table	67.8 ±2.3	69.0 ±4.8			
	(66.2 - 69.4)	(64.9 - 78.8)			
			6	262.125	*FNocEnv
			6	719.458	*MNocPer
19. Dragging of tables	67.5 ±2.0	71.3 ±7.6			
	(64.9 - 71.0)	(64.4 - 98.0)			
			6	295.792	*FMorPer
			16	622.889	*FEvePer
			16	273.691	*SEveEnv
20. Suction intake	66.6 ±2.5	66.0 ±2.7			
	(62.0 - 69.0)	(62.0 - 69.0)			
			7	287.857	*FMorPer
			7	616.873	*MNocPer
21. Running water	66.1 ±2.7	66.2 ±1.6			
	(61.1 - 69.0)	(64.1 - 68.0)			
22. Medical visit	63.5 ±2.1	66.3 ±2.5			
	(61.4 - 65.5)	(62.7 - 70.0)			

M = Monday, F = Friday, S = Sunday. Mor = Morning, Eve = Evening, Noc = Nocturnal, Per = Periauricular, Env = Environmental. dB = decibels. DF = degrees of freedom. F = analysis of variance (ANOVA). Significance *p<0.05; ** *p*(0.001.

## Discussion

The intensity average of daytime noise in the NICU studied is above the standards recommended by the AAP (45 dB), periauricular (64.2 dB) and environmental (63.4 dB), which is also the case for nocturnal levels (35 dB), 63.7 and 63.4 dB periauricular and environmental, respectively.(4) High noise levels originate from sources that generate transitory sudden noise ranging in the periauricular point from 68.1 to 70.9 dB and continuous noise has average magnitudes of 67.6 dB periauricular and 68.7 dB environmental, which surpass the noise limits of the Mexican standards; sudden noise must not be > 60 dB and continuous noise must not be > 45 dB.(15) Similarly, noise level reports in a Mexican hospital always exceeded the recommendations, given that its level of environmental noise (30 cm outside the incubator) was 58.7 dB and 60.9 dB periauricular (within the closed incubator 30 cm from the neonate’s pinna), as observed in a neonatology service in a private hospital in Mexico city,(16) as well as in Brazil in the NU prior to an intervention with the “quiet hour”, presented levels around 70 dB and that after said intervention it was reduced by 20 dB.(10) The same was reported in a study on noise level conducted in Portugal in three NICU; the findings showed levels between 48.7 and 71.7 dB, magnitudes quite similar to the levels in the present study.(11) With respect to the noise level variability in the different days and shifts evaluated, it may be deduced that the loudness is given by activities, type of patient requiring vital support equipment according with their state of complexity, and number of people in the NICU in each institution. According with the critical areas of study within NICU, sector V had lower noise intensity tan the rest, which, although also the types of patients was similar and with vital support equipment present, it is mentioned that it is an area with less transit, favored by characteristics, like sinks further away from the patient at 10-m distance approximately, unlike the 6-m distance in the rest.

Findings in the measurement of environmental and periauricular noise levels in neonate units in the present study show that periauricular noise exceeds environmental noise by at least 0.1 to 2.0 dB; it could be deduced that loudness near the pinna is perceived with greater intensity, added to the fact that care equipment (monitors, nebulizers, suction intake, among others) are at the patient’s headboard, which, when their alarms are activated, produce sudden noises that increase the noise level; their harmful effect could further potentiate the effects of the neonate’s comorbidity and treatments. Exposure to noise ≥ 60 dB has been associated with the potentiation of the effect of ototoxic agents, such as aminoglycosides that can damage the ciliated cells of the ear and produce responses by the preterm babies to high transitory noises that affect principally the cardiovascular system with acceleration, deceleration or biphasic deceleration-acceleration of heart rate and blood pressure; however, the latter does not exceed normality ranges. Results of studies on exposure to noise in NICU are not conclusive with respect to modification of breathing frequency or oxygen saturation. Regarding to the sleep state, which - as known - is fundamental in the neonate’s neurodevelopment, it is affected by noise and provokes states of irritability or crying. It has been noted that establishing the quiet hour produces increased duration of sleep in preterm babies;(7,10) nevertheless, the child returns to the prior state of noise levels and it continues affecting the neonate in the NICU.(4) Moreover, effects of noise have been reported, such as stress, pain, alterations in growth hormone production and, specifically, in the preterm baby somatic adverse effects in sleep, hearing damage, and disorders of emotional development.(17,18)

In relation to noise sources, the study highlights mechanical events that produce greater noise and which due to their nature may be avoided or have their loudness reduced, like handling of formula bottles, alarms of various types, movement of furnishings or their parts, sounds of objects due to falls, placement on a surface, or dragging on the floor. In this respect, maintenance or replacement of furniture, equipment and fixtures should be sought to make environmental and periauricular noise reduction possible.(4)

The highest noise level occurred during the morning shift; similar to that reported in the pre-intervention assessment for noise reduction in the NU in a hospital in Monterrey, Mexico with 59.7 ±5.0 dB, activities,(19) formal interaction (nursing change of shift), as well as informal interaction by the staff (conversation), contribute significantly to the noise level.

As an effect of this study, during the days of noise level evaluation, the hospital staff modified their voice volume, responded immediately to the alarms, and turned off the radio recorder; even so, the results evidence higher limits than those permitted. The nursing staff in the NICU, although sensitized to reduce noise from alarms, is faced with the challenge to constantly respond to alarms, especially those of manual control because their multiple tasks do not allow for this. Due to this, and according with study results, automatic alarms could be used in NICU based on the neonate’s saturation,(20) meaning that an institutional noise reduction policy is required, which implies a permanent program to reduce sound stimuli in the NICU and where the health staff participates comprehensively.

It is worth mentioning that studies have also analyzed the impact of noise on the nursing staff that remains during complete shifts in direct patient care in the UN; the staff attributes the noise level as a significant factor, the manifestation of signals and responses regarding the environment in the UN, especially in the NICU, as the burnout syndrome, tiredness, headache, and mood disorders such as irritability. These conditions become chronic, depending on the amount of time in the NICU, condition a greater amount of errors in professional performance and accidents. The staff possibly confronts this wear with mechanisms, like music during the shift and informal chatting near and far away from the patient that, in turn, raise noise levels, added to the high level of noise making the staff to raise their voice to be heard by other members of the health staff in the NICU; hence patients and staff must be considered in noise reduction programs. In addition, these studies report as an important finding that the nursing staff is not trained in noise reduction strategies and interventions in the NU.(4,11)

Among the study limitations, the study did not manage to determine with specificity the isolated source, only through the sudden rise coinciding during registries during the measurement process. Although an intentioned measurement was carried out of certain noise sources in different areas, day, and schedule, the recording of decibels was quite variable, probably because of existing background noise and the technical part did not have an expert on procedures of acoustic measurements.

It is recommended for noise factors that are preventable to be reduced; the institution and the health staff must favor a safe environment for the recovery and development of neonates at risk, especially preterm babies. It is important to have this hospital policy and have a program and sensitivity campaign and training for noise reduction, as well as provide vital support equipment, quality organizational and architectural factors. Beneficial sounds should be included, such as soft and modulated voices from the parents and from the staff in charge, given that recognizing sounds of human voices favors language development.(12,21) Periodical samplings of the noise levels are suggested to compare if the actions implemented contribute to diminish such.

This study concludes that environmental and periauricular noise in NICU exceed by twice and almost thrice the 45 dB during the day (59.06 to 77.73 dB) and during the night shift (60.8 to 73.5 dB) with respect to 35 dB at night in hospitals, as recommended by the American Academy of Pediatrics. Also, sudden noise levels (67.9 to 70.8 dB) and continuous noise (67.6 to 68.7 dB) exceed the regulating criteria of noise levels in Mexican hospitals within the NICU that must not exceed 60 dB of transitory noises and 45 dB of environmental continuous noise according with the Mexican Official Norm (NOM - 025-SSA3-2013) for the organization and operation of intensive care units. Noise level is higher in the morning shift during the days evaluated. Noise sources are from mechanical origin (alarms) and from human activity, especially conversation by the staff and change of nursing shift.

It is important for the NU staff and specifically the nursing staff to recognize their participation in the production of high levels of noise in this environment, given their 24-h per day permanence and may contribute to improving the acoustic space to care for a highly vulnerable population, like preterm children and others, contribute to improving their own work environment, given that it is known that noisy environments produce stress in the nursing staff and this combination is inversely related with the level of job satisfaction and el chronic wear. Participation must be through application of strategies and actions based on continuous training.
